# A maize protoplast transfection system for studying the biosynthesis of volatile terpenoids

**DOI:** 10.1007/s44297-026-00076-5

**Published:** 2026-05-07

**Authors:** Jinfeng Qi, Mengjing Li, Zhonghua Hu, Runsen Li, Jing Li, Mou Zhang, Canrong Ma, Jianqiang Wu

**Affiliations:** 1 Department of Economic Plants and Biotechnology, Yunnan Key Laboratory for Wild Plant Resources, Kunming Institute of Botany, Chinese Academy of Sciences, Kunming, China; 2 CAS Center for Excellence in Biotic Interactions, University of Chinese Academy of Sciences, Beijing, China; 3 State Key Laboratory of Plant Diversity and Prominent Crops, Beijing, China

**Keywords:** Maize protoplast, *ZmTPS10*, (*E*)-β-Farnesene, (*E*)-α-Bergamotene

## Abstract

Volatile terpenoids are produced by almost all plants. They often function as important defensive secondary metabolites. Currently, research aimed at elucidating the biosynthesis of maize terpenes is limited. Thus, a rapid and efficient platform for identifying genes involved in the synthesis/regulation of maize volatile terpenes is highly desirable. Through optimization of various conditions, including light, substrate accumulation, nutrient and ionic composition of the culture medium, culture vessel size and conditions, organic solvent extraction, and promoter selection, we established a maize protoplast transfection system for analyzing the biochemical functions of terpene synthases (TPSs) and the functions of regulatory factors, such as transcription factors. Specifically, dim light-cultured maize seedlings are first treated with methyl jasmonate, followed by preparation of protoplasts and transfection of target genes driven by the *ubiquitin* promoter. The transfected protoplasts were cultured overnight in glass headspace tubes. Maize protoplasts were extracted by n-pentane and concentrated, and after being heated, the volatile terpenes were absorbed by solid-phase microextraction and analyzed on a gas chromatograph. This system was successfully used to detect the products of *ZmTPS10*, (*E*)-β-farnesene and (*E*)-α-bergamotene and to analyze the function of the transcription factor *ZmMYC2a* in regulating the production of (*E*)-β-farnesene and (*E*)-α-bergamotene. Our system provides a rapid and efficient platform for studying the synthesis and regulation of maize terpene volatiles and can also be adopted for other plant species.

**Supplementary Information:**

The online version contains supplementary material available at 10.1007/s44297-026-00076-5.

## Introduction

Plants and insects have coevolved for more than 400 million years [1]. Almost all plants are attacked by insects, and through long-term evolution, plants have acquired diverse resistance mechanisms against insects [2]. Plants use direct and indirect defenses to resist insects [3]. Direct defenses are toxins, antifeedants, physical barriers, or other plant defensive traits that deter insects, and indirect defenses are those plants use to attract predators of insects [4]. The molecular mechanisms of plant resistance to insects have been intensively studied in dicotyledonous plants such as Arabidopsis (*Arabidopsis thaliana*) and tomato (*Solanum lycopersicum*). However, the insect resistance mechanisms in monocots, including maize, remain poorly understood [5]. Maize is a staple food crop cultivated in most countries worldwide and holds an extremely important position in agriculture. Many insects infest maize, including *Mythimna separata*, *Spodoptera frugiperda*, *Spodoptera exigua, Helicoverpa armigera*, and *Rhopalosiphum maidis*, causing large economic losses [6], and consequently, maize deploys defense-related responses at the levels of the transcriptome, proteome, and metabolome, although the underlying mechanisms remain largely unknown [7]. Maize was the first plant discovered to specifically perceive FACs (fatty acid‒amino acid conjugates) present in insect oral secretions and therefore activate direct and indirect defenses [8], including benzoxazinoids and various volatile and nonvolatile terpenoids [6].

Terpenoids (also called isoprenoids) are the largest and most structurally diverse class of natural compounds, encompassing over 40,000 identified structures across all kingdoms of life and serving essential roles in plant growth, development, and interactions with the environment [9, 10]. They are derived from two universal five-carbon building blocks, isopentenyl diphosphate (IPP) and its isomer dimethylallyl diphosphate (DMAPP), which are produced via two compartmentalized pathways: the mevalonate (MVA) pathway in the cytoplasm and the 2-C-methyl-d-erythritol 4-phosphate (MEP) pathway in plastids [11]. In the MVA pathway, acetyl-CoA is sequentially condensed and reduced to mevalonate and then phosphorylated and decarboxylated to yield IPP/DMAPP, primarily supplying precursors for sesquiterpenoids, triterpenoids, and sterols; the MEP pathway, operating in plastids, uses pyruvate and glyceraldehyde-3-phosphate to generate IPP/DMAPP for hemiterpenoids, monoterpenoids, diterpenoids, carotenoids, and hormones such as gibberellins [9, 11]. Prenyltransferases condense these C₅ units into geranyl diphosphate (GPP, C10), farnesyl diphosphate (FPP, C15), and geranylgeranyl diphosphate (GGPP, C20), which are then cyclized and modified by terpene synthases (TPSs) and cytochrome P450 enzymes to produce the vast diversity of terpenoid structures [9].

All tissues of maize can release terpenoid volatiles. Although under normal conditions, the terpene volatiles in maize are often low, the composition and released levels of volatiles vary depending on the maize variety, developmental stage, organ, and biotic or abiotic stress encountered [12]. The maize genome encodes approximately 30 terpene synthase (TPS) genes [13], and approximately half of the genes have relevant reports on their catalytic products [12]. A single TPS may catalyze different substrates to produce multiple products. For example, maize TPS1 (GRMZM2G049538) can catalyze the synthesis of the acyclic monoterpene volatiles linalool and geraniol using GPP as the substrate and volatile sesquiterpenes such as (*E*)-β-farnesene (EBF), (*E*, *E*)-farnesol, and (*E*)-nerolidol using FPP as the substrate [14]. Terpene volatiles may also be formed through further oxidation, dehydrogenation, acylation, or other cytochrome P450-mediated modifications. For example, the products of TPS2 (GRMZM2G046615), (*E*)-nerolidol and (*E*, *E*)-geranyllinalool are converted by the P450 monooxygenases CYP92C5 (GRMZM2G102079) and CYP92C6 (GRMZM2G139467) into (3*E*)−4,8-dimethyl-1,3,7-nonatriene (DMNT) and (*E*, *E*)−4,8,12-trimethyltrideca-1,3,7,11-tetraene (TMTT) [15], and DMNT and TMTT are volatile homoterpenes that are produced in maize under biotic stresses and are typically released into the headspace of damaged maize tissues [16].

Many terpene volatiles can attract parasitic or predatory insects to attack herbivorous insects. Previous analysis indicated that maize volatile terpenoids likely play important roles in maize resistance to insects as indirect defenses. It was found that when fed *S. exigua*, maize seedlings release large amounts of volatiles, including linalool, DMNT, indole, (*E*)-α-bergamotene (EAB), EBF, (*E*)-nerolidol, and TMTT, which attract the parasitoid wasp (*Cotesia marginiventris*) of *S. exigua* [16]. Linalool attracts the parasitic wasp *Campoletis chlorideae*, a natural enemy of *M. separata* [17]. (*E*)-β-Caryophyllene not only attracts the parasitoid wasp *Cotesia sesamiae,* which preys on the spotted stem borer *Chilo partellus* on leaves [18], but numerous studies have also reported its synthesis and release through roots, acting as a crucial volatile for attracting parasitic or predatory natural enemies to protect maize from the western corn rootworm (*Diabrotica virgifera virgifera*) underground [19]. Herbivory-induced terpene volatiles may also function as direct plant defenses against insect herbivores. Recent research has indicated that DMNT can directly harm the lepidopteran insect *Plutella xylostella* by damaging its peritrophic matrix [15].

Investigating the function and products of volatile synthesis genes is commonly achieved through prokaryotic expression of TPSs [20]. However, there are several drawbacks of these systems, such as a) inability to perform complex post-translational modifications (PTMs), given that prokaryotes such as *Escherichia coli* lack the machinery for eukaryotic-specific PTMs, which could be important for the biological activity, stability, and solubility of some TPSs; b) formation of inclusion bodies: high-level expression of recombinant proteins often leads to accumulation of misfolded proteins as insoluble aggregates (inclusion bodies); and c) protein misfolding and aggregation: the cellular environment of prokaryotes may lack specific chaperones or have an inefficient secretion system, leading to incorrect protein folding, aggregation, and loss of activity, even for soluble proteins [21, 22]. To overcome these disadvantages of prokaryotic expression systems, genetically modified plants are often needed for functional analysis of TPSs, and in terms of maize, maize TPS mutants have been used for studying TPSs [19, 23]. Owing to the long cycle and high cost of maize genetic transformation, there is an urgent need to develop rapid and efficient methods for identifying and screening genes involved in maize terpene volatile synthesis and regulation.

Protoplasts are plant cells whose cell walls are removed by enzyme digestion, and exogenous DNA, RNA, and proteins can be relatively easily delivered into protoplasts through physical or chemical methods [24]. For example, DNA can be transferred into protoplasts by polyethylene glycol (PEG)-mediated transfection or electroporation, enabling transient expression of genes of interest within a short period of time, often 24–48 h. Maize protoplasts have been used for gene function verification, promoter activity analysis, and protein interaction studies, and maize protoplasts can also be used for studying the biosynthesis and regulation of nonvolatile secondary metabolites, such as benzoxazinoids [25–28]. However, whether maize protoplasts can be used to study TPS genes has not been reported to date. Here, using *ZmTPS10* as the target gene, through optimization, we established a protocol of protoplast transfection for studying maize TPS genes. This platform is expected to provide a powerful tool for maize functional gene research, and this method could also be used for functional characterization of TPSs from other species using protoplasts isolated from maize or other species.

## Results

### Plant growth conditions- light promotes the biosynthesis of volatile terpenes

**Fig. 1:**
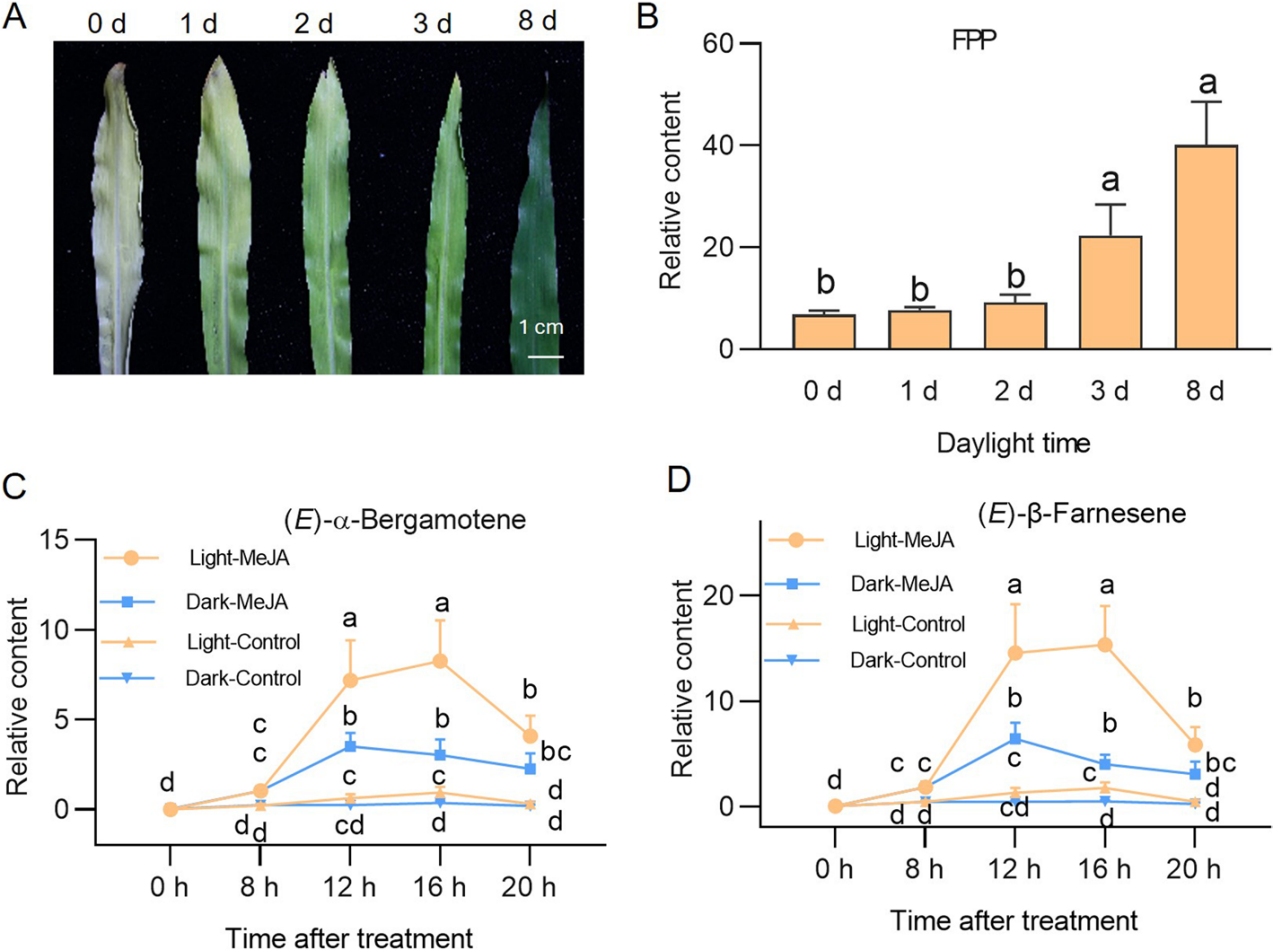
Light promotes volatile accumulation. **A** Photograph of maize leaves cultured under different light conditions. From left to right: the second leaf of 12-day-old maize seedlings, which were grown in darkness for 12, 11, 10, 9, or 4 days before receiving 0, 1, 2, 3, or 8 days (1, 2, 3, 8 d) of relatively dim light (light intensity 4.25 μmol⋅m^−2^⋅s.^−1^, 16 h of light/8 h of dark). **B** Relative contents of FPP in leaves of maize seedlings treated with different light exposure times. The leaves shown in (A) were treated with 1 mM MeJA for 16 h before they were harvested for the measurement of FPP. **C-D** Relative contents of (*E*)-α-bergamotene and (*E*)-β-farnesene in greenish (12 d) or etiolated (0 d) seedlings induced by MeJA treatment. The seedlings were either treated with 1 mM MeJA or mock treated (control), and after the indicated times, the second leaves were harvested for quantification of (*E*)-α-bergamotene and (*E*)-β-farnesene. Different letters indicate significant differences between groups (one-way ANOVA followed by Duncan’s multiple range test, n = 4–5; *p* < 0.05; data are mean + SE)

Previously, when we optimized maize protoplast transfection for studying the biosynthesis and regulation of benzoxazinoids, etiolated seedlings were used because protoplasts isolated from normal green maize seedlings are prone to rupture during transfection. To evaluate whether protoplasts isolated from etiolated seedlings are suitable for studying TPSs, we first used protoplasts from maize seedlings grown under different light conditions. The maize seedlings were cultivated under complete darkness (etiolated seedlings) for 12 days, or they were grown in darkness for 11, 10, 9, or 4 days before being transferred to a greenhouse with 16 h of light/8 h of darkness, receiving 1, 2, 3, or 8 days of relatively dim light (4.25 μmol⋅m^−2^⋅s^−1^) (Fig. 1A). Given that jasmonic acid (JA) signaling often activates the biosynthesis of secondary metabolites, including terpenes [29], the seedlings were treated with methyl jasmonate (MeJA), and the contents of the sesquiterpene precursor FPP as well as EAB and EBF in the leaves were measured. The FPP content increased by 3.3- and 5.9-fold in maize after receiving 3 and 8 days of weak light, respectively, compared with that in the completely etiolated leaves (Fig. 1B), indicating that light and MeJA treatment both activate FPP biosynthesis, especially in the leaves treated with 8 days of light (those that received scattered light for 8 days, henceforth referred to as greenish seedlings). Additionally, after MeJA treatment, the relative levels of EAB and EBF in seedlings were much higher in the greenish seedlings than in the etiolated seedlings (Fig. 1C, D). For example, at 16 h post-MeJA treatment, EAB and EBF released from the greenish seedlings were 2.7-fold and 3.8-fold greater, respectively, than those released from the etiolated seedlings (Fig. 1C, D). Thus, light conditions promote the biosynthesis of volatile terpenes, and seedlings that received 8 days of dim light will be used for the following experiments.

### Selection of leaf parts

**Fig. 2:**
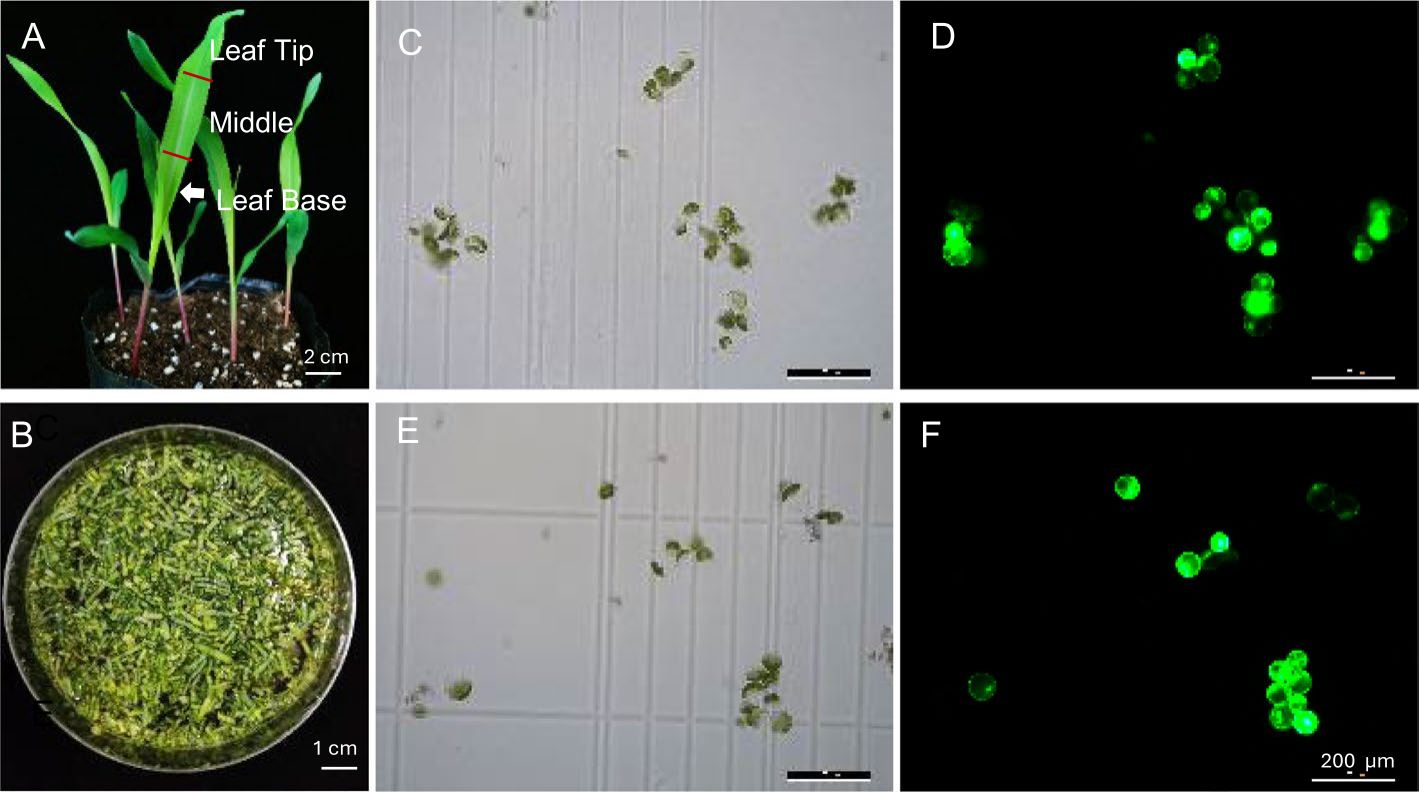
Isolation and transfection of protoplasts from greenish maize leaves. **A** Photograph of the tip, middle, and base parts of the second leaf from a 12-day-old greenish seedling. **B** Enzymatic lysis of maize leaf segments. **C-F** Protoplasts from the leaf base (**C**, **D**) and middle (**E**, **F**) 16 h after transfection with *eGFP*. **C** and **E**, bright field images; **D** and** F**, fluorescence images

To optimize the conditions of maize protoplast isolation for analyses of TPSs, we first determined which part of leaves is optimal. The base, middle, and tip of the second leaves were excised for protoplast isolation (Fig. 2A, B). The protoplasts prepared from the leaf base (Fig. 2C, D) and middle regions (Fig. 2E, F) exhibited good integrity and high transfection efficiency (over 90%) after transfection of a plasmid carrying *eGFP* (enhanced green florescence protein). However, a portion of the protoplasts prepared from leaf tips ruptured after transfection. Consequently, the middle and base regions of greenish seedling leaves were selected for subsequent protoplast preparations.

### Selection of organic solvents for extraction of volatile terpenes

**Fig. 3:**
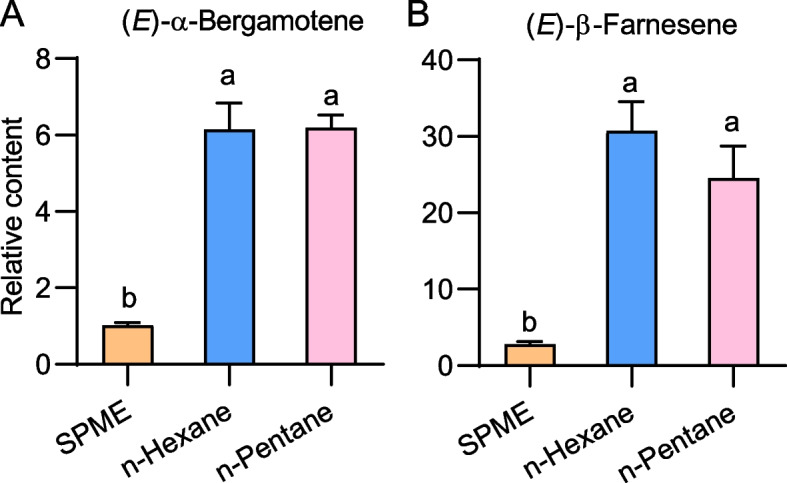
Extraction of volatile terpenes from maize protoplasts. Greenish maize leaves were sprayed with MeJA, and protoplasts were isolated. The headspace of protoplasts was directly used for SPME, or the protoplasts were extracted with n-hexane and n-pentane, and after being concentrated and heated, the headspace was extracted with SPME. The SPME samples were analyzed on a gas chromatograph for quantification of (*E*)-α-bergamotene (**A**) and (*E*)-β-farnesene (**B**). Different letters indicate significant differences between groups (one-way ANOVA followed by Duncan’s multiple range test, n = 4; *p* < 0.05; data are mean + SE)

We compared the efficacy of volatile compound detection using direct overnight SPME adsorption versus overnight protoplast culture followed by extraction with different organic solvents and subsequent SPME analysis. The results showed that the effectiveness of SPME adsorption after extraction with n-hexane or n-pentane was much greater than that of direct overnight SPME adsorption (Fig. 3). Since n-pentane is less toxic than n-hexane and more volatile, in subsequent experiments, protoplasts were extracted with n-pentane, followed by SPME adsorption.

### Optimization of protoplast culture medium and cultivation conditions

**Fig. 4:**
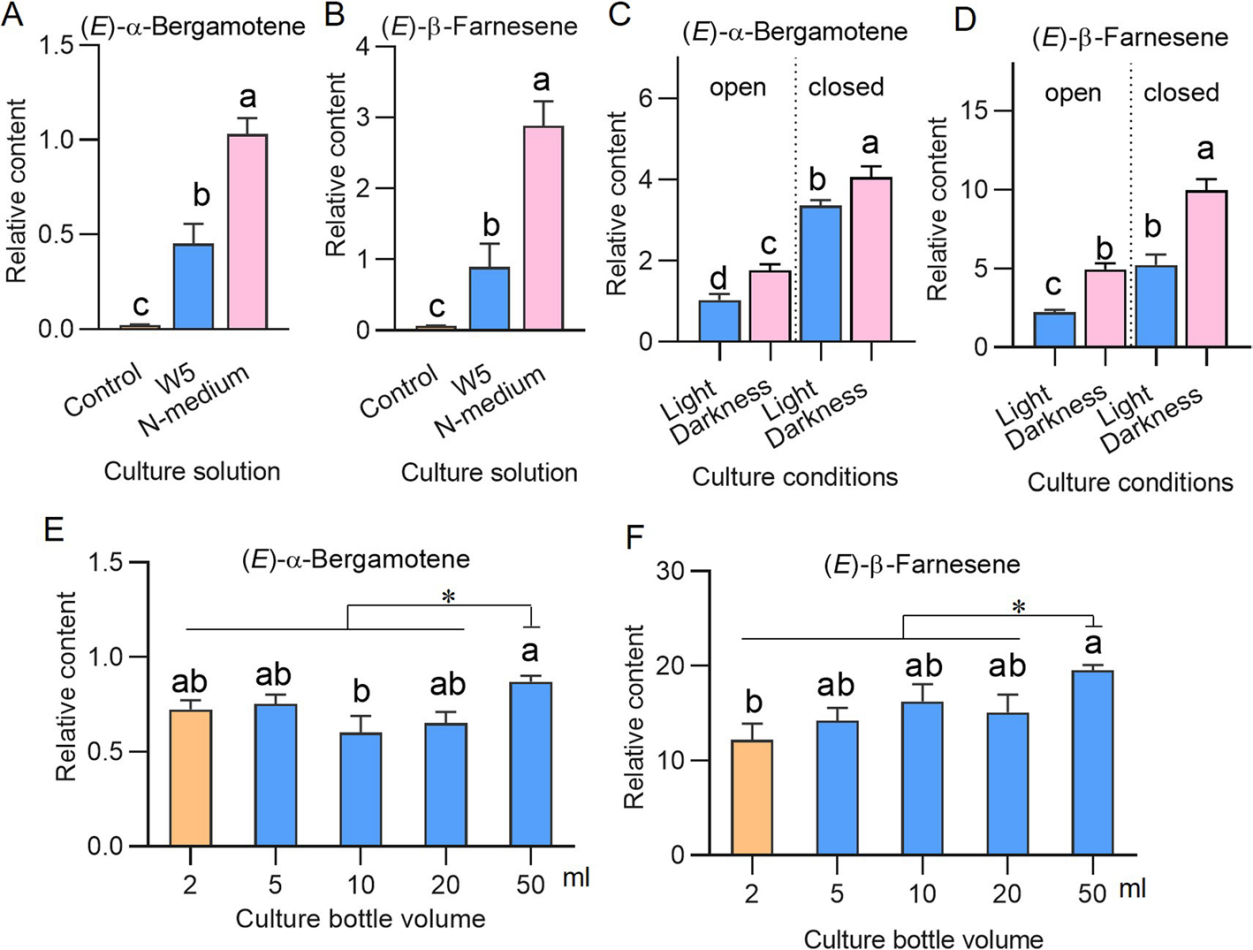
Effects of culture conditions on the levels of terpene volatiles produced in maize protoplasts. Maize protoplasts were isolated from greenish leaves that were not pretreated with MeJA. **A, B** Relative contents of (*E*)-α-bergamotene (**A**) and (*E*)-β-farnesene (**B**) in protoplasts cultured for 0 h (control, in MMG buffer) or 16 h in W5 solution or N-medium. **C**, **D** Relative contents of (*E*)-α-bergamotene (**C**) and (*E*)-β-farnesene (**D**) in protoplasts cultured under different conditions. Protoplasts were provided with or without light and cultured in 20-mL closed or open tubes. **E, F** Relative contents of (*E*)-α-bergamotene (**E**) and (*E*)-β-farnesene (**F**) in protoplasts cultured in closed headspace tubes with different volumes. The 2-mL tube contained 0.5 mL of protoplasts, while in the other tubes, an equal number of protoplasts was diluted to a total volume of 2 mL. Different letters indicate significant differences between groups (one-way ANOVA followed by Duncan’s multiple range test). “*” represents significant differences between samples detected in 50-ml tubes and each other (Student’s *t*-test, n = 4, *p* < 0.05). Data are mean ± SE

To investigate the effects of nutrients in the culture medium on the synthesis of volatile terpenoids in maize protoplasts, we added 10 mM sucrose, 20 mM glucose, and 4.43 g/L MS to the culture media (nutrient-rich medium, N-medium). Maize protoplasts (greenish seedlings were first treated with MeJA, followed by protoplast preparation) were cultivated in W5 solution [26], which contains no nutrients or N-medium (Table S1). We found that protoplasts cultured in N-medium accumulated higher levels of EAB and EBF than those cultured in W5 (Fig. 4 A, B), indicating that adding nutrients to the culture medium can provide the energy required for cellular physiological activity. Therefore, N-medium was further used as the optimal culture medium for subsequent experiments.

Our previous experiment demonstrated that light positively regulates the synthesis of EAB and EBF in maize leaves (Fig. 2B). However, the effect of light on the synthesis of volatile terpenoids in protoplasts remains unclear. We established two culture conditions: light (4.25 μmol⋅m^−2^⋅s^−1^) and dark. Considering that oxygen levels may affect the accumulation of volatiles, two more experimental conditions were included: open and sealed containers. It was found that in the protoplasts cultured under dark and sealed conditions, the contents of EAB and EBF were the highest (Fig. 4C, D). The contents of EAB and EBF in the light-treated groups were always lower than those in the dark-treated groups (Fig. 4C, D). Additionally, 2, 5, 10, 20, and 50 mL headspace tubes were used for culturing the protoplasts, and we found that the levels of EBF increased when the tube volume increased: the EBF contents in the 50-mL tubes were 60% greater than those in the 2-mL tubes (Fig. 4E, F). Thus, 50-mL headspace tubes were used for protoplast culture.

### Promoter selection

**Fig. 5:**
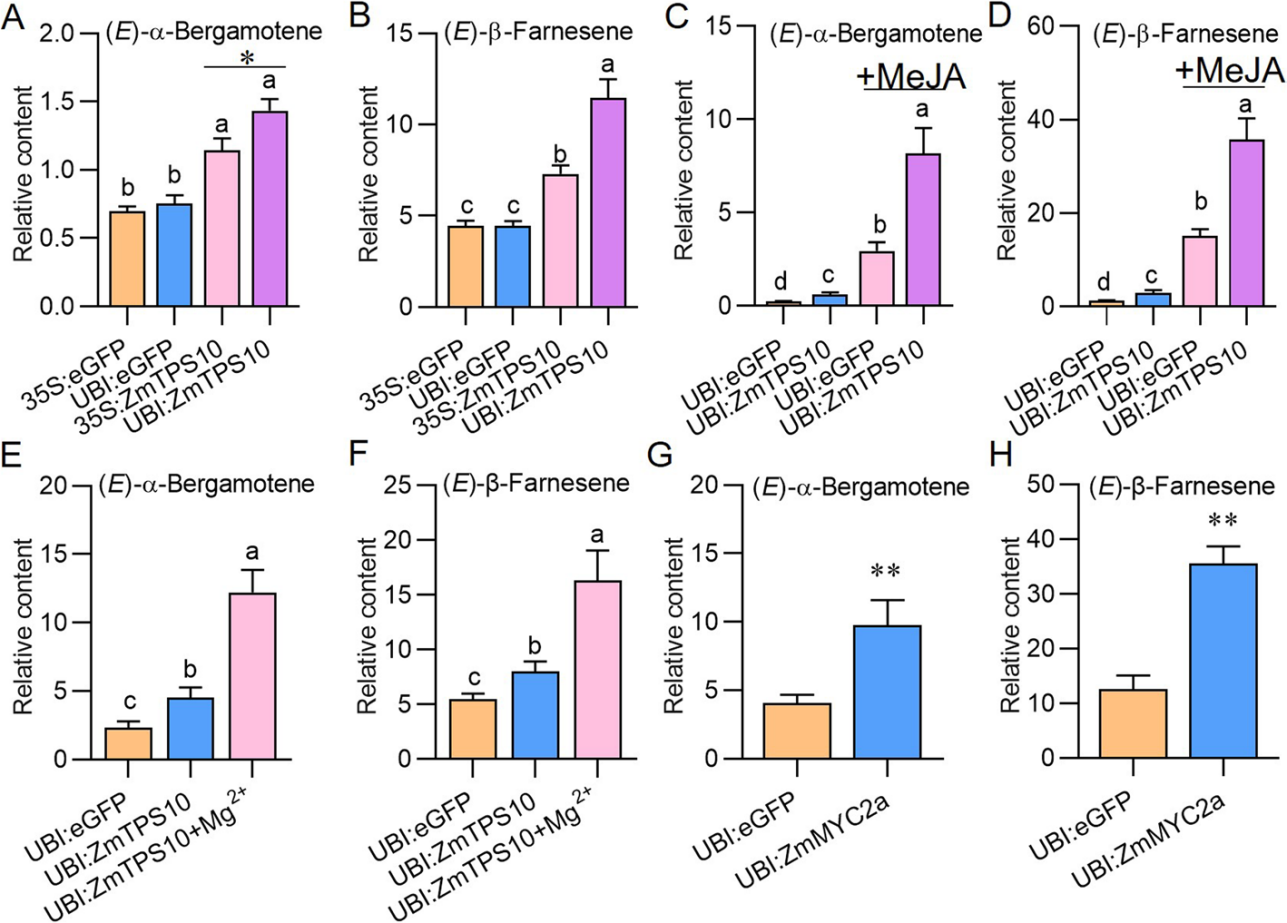
Effects of promoters, MeJA, and Mg^2+^ on the production of terpene volatiles in maize protoplasts. **A-D** Relative contents of (*E*)-α-bergamotene (**A**, **C**) and (*E*)-β-farnesene (**B**, **D**) in protoplasts transfected with *eGFP* or *ZmTPS10* driven by the 35S or UBI promoter. Maize protoplasts were isolated from greenish leaves that were not pretreated with (**A**, **B**) or pretreated with (**C**, **D**) MeJA. **E**, **F** Relative contents of (*E*)-α-bergamotene (E) and (*E*)-β-farnesene (**F**) in maize protoplasts transfected with *eGFP* or *ZmTPS10* with or without Mg^2+^. **G**, **H** Regulation of the *ZmMYC2a* transcription factor on the levels of (*E*)-α-bergamotene (**G**) and (*E*)-β-farnesene (**H**). Maize protoplasts were transfected with plasmids expressing *eGFP* or *ZmMYC2a,* and the levels of (*E*)-α-bergamotene and (*E*)-β-farnesene were analyzed. Different letters indicate significant differences between groups (one-way ANOVA followed by Duncan’s multiple range test); “*” represents differences between the two indicated groups, Student’s *t* test, n = 4–7; (* *p* < 0.05, ** *p* < 0.01). Data are mean ± SE

The *ubiquitin* (UBI) and cauliflower mosaic virus 35S (CaMV35S) promoters are both widely used to drive target genes in protoplasts [30]. To assess the applicability of the two promoters, *ZmTPS10* or *eGFP* (as a control) driven by CaMV35S and UBI was transfected into maize protoplasts, as the *ZmTPS10* gene in maize has been reported to catalyze the production of EAB and EBF in vitro and to play an important role in attracting parasitic wasps [31]. In protoplasts transfected with *UBI:ZmTPS10*, the relative levels of EAB and EBF were greater than those in the other groups (Fig. 5A, B). Therefore, the UBI promoter was used in subsequent experiments.

### MeJA application and the addition of Mg^2+^

In the absence of MeJA treatment, small levels of EBF and EAB were detected in maize protoplasts transfected with *eGFP* (Fig. 5C, D). In contrast, the accumulation of EAB and EBF in the *eGFP*-transfected protoplasts, which were isolated from MeJA-treated leaves, increased more than 10 times (Fig. 5C, D). Importantly, *ZmTPS10* overexpression further elevated the levels of both compounds. Thus, MeJA treatment of leaves can markedly boost EAB and EBF accumulation in protoplasts isolated from these leaves, thereby improving the detection limit and reliability and reducing the number of protoplasts required for experiments.

Previous studies have shown that ZmTPS10 enzyme activity requires the cofactor Mg^2+^ [12, 32]. All TPS genes possess a highly conserved Asp-rich DDxxD domain, which is associated with Mg^2+^ binding [12]. Therefore, we investigated whether the presence of Mg^2+^ in the culture medium affects the accumulation of volatile terpenes in protoplasts transfected with *ZmTPS10*. Indeed, the addition of Mg^2+^ (10 mM) to the culture medium increased the contents of EAB and EBF by 2.7- and 2.0-fold, respectively (Fig. 5E, F), indicating that Mg^2+^ can increase the enzymatic activity of ZmTPS10 in maize protoplasts.

### Using maize protoplasts to study the regulation of volatile terpenes

Our previous study revealed that the transcription factor *ZmMYC2a* can positively regulate the synthesis of EAB and EBF [26]. After overexpressing *ZmMYC2a* in maize protoplasts, we found that the levels of EAB and EBF were much higher than those in the control (Fig. 5G, H), indicating that the protoplast-based volatile detection system can also be used to study the roles of transcription factors in the regulation of volatile terpenes.

## Discussion

Both herbivore insect feeding and oviposition can induce the release of volatiles from plants, mainly terpenoids, green leaf volatiles (GLVs), aromatic compounds such as indoles, methyl salicylic acid, and various other compounds [4]. Insect-induced volatiles are not only produced in the leaves of plants and released into the atmosphere but also generated in the roots and released into the rhizosphere [19]. The composition of volatiles induced by insect feeding varies significantly depending on the plant species, the species of feeding insect, the feeding site, and even the time of feeding. For example, feeding by the larvae of the tobacco budworm *Heliothis virescens* induces several volatile compounds that are released only at night and exhibit high repellency to female moths, helping them accurately locate suitable oviposition sites (where host plants were infested by other caterpillars) and instead locate areas with sufficient food for their offspring [33]. Insect-induced volatiles primarily serve two functions: 1) the natural enemies of the feeding insects are attracted for predation or parasitism, thereby acting as an indirect defense [6]; 2) they are perceived by neighboring plants or undamaged tissues, enabling faster and stronger defense responses when the plant is attacked again by insects, a phenomenon known as “defense priming” [34, 35]. Maize has often been used as a model plant for studying insect-induced volatiles. Many critical discoveries, such as herbivore-induced plant volatiles attracting natural enemies of pests to mediate indirect plant defense [16], green leaf volatiles [36] and indoles [37] priming plants to induce antiherbivore defenses and the observation that younger leaves exhibit stronger systemic antiherbivore capabilities in response to green leaf volatiles than older leaves [38], were all first reported in maize. However, the specific products of TPSs in maize, their respective ecological functions, and many other scientific questions remain to be explored.

Transfection of protoplasts is relatively simple and rapid. Taking advantage of this property, Arabidopsis protoplasts have been widely used for studying protein localization, protein–protein and protein‒RNA/DNA interactions, and protein biochemical functions [39]. Artificial microRNAs (amiRNAs) and clustered regularly interspaced short palindromic repeats (CRISPR)/Cas9 are used for silencing and knocking-out target genes, respectively, and Arabidopsis and maize protoplasts have been used for screening the optimal amiRNA- or CRISPR/Cas9-target sites [30, 40, 41]. Recently, cleavage under targets and tagmentation (CUT&Tag) has also been developed for protoplasts [42]. Previously, we developed a maize protoplast transfection system for studying the biosynthesis and regulation of maize benzoxazinoids, which are not volatile metabolites. Here, we established a maize protoplast system for determining the biochemical functions of TPSs. This refined system overcomes the inherent challenges of volatile analysis and offers several distinct advantages over conventional approaches.

### The dual paradox of “light” and the selection of cell developmental stages

In plant protoplast related research, etiolated seedlings are the preferred source for isolating highly stable protoplasts [25], which is primarily attributed to the distinct cellular architecture established during skotomorphogenesis, characterized by a prominent central vacuole [43]. This structural configuration maintains high turgor pressure, which is crucial for preserving plasma membrane integrity and conferring resistance against mechanical stress—such as centrifugation and pipetting—following enzymatic cell wall digestion.

However, this study focuses on terpenoid biosynthesis, a process where light acts as a critical environmental cue for inducing the accumulation of terpene precursors (e.g., FPP). This presents a "light paradox": while complete darkness optimizes protoplast viability, it restricts the accumulation of secondary metabolites. To resolve this, we implemented a dim light pretreatment strategy to delicately balance cellular stability with metabolic competence. We postulate that this low-intensity irradiation is sufficient to trigger initial plastid development and activate precursor synthesis pathways but insufficient to induce the proliferation of fragile, mature chloroplasts. Consequently, cells retain their large vacuolar morphology while acquiring the metabolic potential required for terpenoid synthesis.

Furthermore, this finding elucidates why leaf tip tissue is unsuitable for protoplast isolation. The maize leaf functions as a spatiotemporal developmental continuum, exhibiting a gradient from the basal meristem (cell division zone) to the distal tip (mature zone). The leaf tip represents a late developmental stage where the central vacuole has typically fragmented into smaller vacuoles, and the cytoplasm is densely packed with chloroplasts. This structural reorganization leads to a significant decline in osmotic stability and mechanical strength, rendering the cells highly susceptible to rupture during manipulation [44].

### Metabolic precursor bottlenecks and the “in planta priming” strategy

In this study, the strategy of "feeding" protoplasts via exogenous MeJA treatment of mother plants offers distinct operational and economic advantages. This "in planta priming" approach leverages the robust primary metabolic network of the intact plant to enrich precursors, thereby circumventing the need for direct supplementation of expensive and unstable FPP in the protoplast culture medium. Unlike heterologous systems (such as *E. coli* or yeast), maize protoplasts serve as a "homologous chassis." They possess maize-specific cofactor environments, membrane systems, and post-translational modification machinery. Consequently, data derived from this system exhibit higher physiological fidelity when characterizing maize gene functions and metabolic pathways.

### From single genes to complex networks: system versatility and expansion potential

The system established in this study extends beyond single-gene functional characterization, offering a versatile platform for the reconstruction of complex signaling pathways. Capitalizing on the high efficiency of multi-plasmid co-transfection in protoplasts, we can simulate and resolve intricate defense signaling networks (e.g., Receptor → Kinase → Transcription Factor → Structural Gene). This "pathway reconstruction" strategy is pivotal for elucidating the mechanisms by which plants integrate environmental signals to initiate metabolic reprogramming.

Crucially, this system holds irreplaceable value in monocot (particularly graminaceous crop) research. Given the recalcitrance of crops such as maize, wheat, and sorghum to genetic transformation—characterized by long cycles and unique cell wall compositions (e.g., silicification)—this method provides an efficient "shortcut" for gene function studies. Looking forward, by optimizing osmotic regulators (e.g., mannitol/sorbitol), enzymatic cocktails (cellulase/macerozyme concentrations), and ion conditions (Mg^2^⁺), this system is poised for extension to other non-model graminaceous species, serving as a vital tool for crop synthetic biology.

## Materials and methods

### Plant materials, protoplast isolation and transfection

The maize (*Zea mays*) inbred line KN5585 was used in this study. The etiolated or greenish seedlings (receiving 4.25 μmol·m⁻^2^·s⁻^1^ light) were used for protoplast isolation. For MeJA treatment, 12-day-old maize seedlings were fully sprayed with 1 mM MeJA solution (containing 0.01% ethanol and 0.05% Tween 20) for 8 h, and plants treated with solvent (containing 0.01% ethanol and 0.05% Tween 20) were used as the control. For protoplast isolation and transfection, the second leaves of 12-day-old greenish seedlings were cut into 1 mm-wide slices and incubated in an enzyme solution as described previously [26]. The released protoplasts were filtered through a 40-μm cell strainer, and the filtrate was centrifuged at 100 × g for 6 min at RT (room temperature), followed by resuspension and 1 h of incubation in W5 buffer on ice, which allowed the protoplasts to settle by gravity. The protoplasts were then resuspended at a concentration of 5 × 10^5^ cells/mL in MMG buffer [26]. The plasmid (1 μg/μL, 50 μg) was then mixed with 500 μL of protoplasts in a 10-mL tube, followed by the addition of 550 μL of PEG-Ca^2+^ solution and gentle mixing. After 15 min, 2.2 mL of W5 buffer was gently added, and the protoplasts were centrifuged at 100 × g for 3 min at RT. The supernatant was discarded, and the protoplasts were resuspended in 2 mL of N-media (2 mM MES [pH 5.7], 5 mM KCl, 125 mM CaCl_2_, 154 mM NaCl, 10 mM sucrose, 20 mM glucose, 4.43 g/L MS) in a 50-mL glass vial (Dikma, cat. no. 52509) with a sealed lid and incubated overnight at 28 °C for 14–16 h. Transfection efficiency was monitored under a fluorescence microscope (Leica DM5500 B) by calculating the percentage of fluorescent protoplasts that were transfected with *eGFP*.

### Plasmid construction

The coding regions of *ZmTPS10* and *ZmMYC2a* were amplified from cDNA synthesized from total RNA isolated from maize leaves and cloned and inserted into the pM999 vector (kindly provided by Dr. Yongzhong Xing from Huazhong Agricultural University). The UBI promoter was amplified from maize genomic DNA and used to replace the original 35S promoter via the homologous recombination method (ClonExpress® II, Vazyme). All primers are listed in Table S2.

### Quantification of volatile terpenes

The quantification of volatiles was performed via a SPME fiber (100 μm polydimethylsiloxane; 57,342-U; Supelco, USA) according to a previous study [26]. Briefly, after transfection and 14–16 h of overnight incubation in a 50-mL glass headspace tube, 5 mL of organic solvent (n-hexane or n-pentane, containing 100 ng of 1-undecanol as the internal standard) was added. Following vigorous extraction on a vortex (2000 rpm, 10 min) and centrifugation (12,000 g, 10 min), the organic phase was collected and concentrated by evaporation to approximately 200 μL under ambient conditions, which was transferred to a 2 mL chromatography vial and evaporated to a final volume of 10–20 μL. The chromatography vial was then capped, and an SPME adsorption fiber was inserted to collect the volatile compounds at 65 °C for 50 min followed by analysis on the gas chromatograph as described previously [26]. Gas chromatographic analysis was performed using a Shimadzu GC-2014 system equipped with an SH-Rtx-5 capillary column (30.0 m × 0.25 mm i.d., 0.25 μm film thickness). The injector temperature was maintained at 250.0 °C, and samples were introduced in splitless mode with an injection time of 2.0 min. High-purity nitrogen (N₂) was used as the carrier gas in constant linear velocity mode with a total flow rate of 4.0 mL/min and a purge flow rate of 3.0 mL/min. The oven temperature program was initiated at 45.0 °C (held for 2.0 min), ramped at 6.0 °C/min to 150.0 °C (held for 3.0 min), and finally increased at 20.0 °C/min to 280.0 °C (held for 2.0 min). The detector temperature was set at 285.0 °C. The SPME fiber was desorbed in the injection port for 3 min prior to analysis to ensure complete release of the adsorbed volatiles.

### Experimental setup for adsorption methods and solvent selection

To extract and detect volatile terpenes from maize protoplasts, we lysed protoplasts isolated from the second leaves, which were treated with MeJA for 8 h. The protoplasts isolated from greenish seedlings were first kept in 20-mL headspace glass vials (2 mL of protoplasts for each vial, 15 vials), which were sealed by caps with polytetrafluoroethylene-lined silicon septa, incubated in the dark at 25 °C and divided into three groups (five vials each) to compare different extraction methods: 1) for one group, we used the “overnight direct adsorption” method, where an SPME (solid-phase microextraction) adsorption fiber was inserted into the glass tube for simultaneous incubation and adsorption for 16 h; 2) in the other two groups, after incubation for 16 h, 5 mL of n-pentane or n-hexane was used to extract volatiles, and after vortexing and centrifugation, the organic solvents were concentrated under ambient conditions to approximately 200 µL, which were transferred to 2-mL liquid chromatography vials and further evaporated to approximately 20 µL. The vials were then sealed and heated at 65 °C to evaporate the volatile compounds to the headspace, and SPME adsorption tips were inserted to collect volatiles for analysis on a gas chromatograph (GC).

### Quantification of FPP

Five biological replicates of leaf samples were prepared. Approximately 150 mg of fresh leaf tissue was collected per replicate, flash-frozen in liquid nitrogen, ground to a fine powder, and stored at –80 °C until analysis. To each powdered sample, 1 mL of extraction solution (70% methanol with 10 mM ammonium hydroxide) was added, followed by vortex mixing for 10 min. The mixtures were centrifuged at 4 °C for 10 min at maximum speed; the resulting supernatants were transferred to glass vials and subsequently analyzed by HPLC–MS/MS using an LCMS-8040 system (Shimadzu). Chromatographic separation was performed on an LC-20AD liquid chromatograph (Shimadzu) equipped with a Shim-pack XR-ODS III column (1.6 μm particle size, 75 × 2 mm i.d.). Samples (10 μL) were injected at a flow rate of 0.3 mL min⁻^1^. The mobile phase consisted of solvent A (5 mM ammonium bicarbonate) and solvent B (acetonitrile), applied in a gradient elution program for both separation and quantification.

### MeJA treatment

We found that MeJA application enhanced the synthesis of FPP (Fig. 2 B), which is the substrate for sesquiterpenoids, such as EBF and EAB. Considering that MeJA application is simple and cost-effective, while the substrate FPP is expensive and is prone to hydrolysis, we investigated whether MeJA application to plants could improve the detection of volatile terpenes in transfected protoplasts. Twelve-day-old greenish seedlings were sprayed with 1 mM MeJA, and the control group was sprayed with buffer. Eight hours after treatment, protoplasts were isolated and used for subsequent transfection.

### Statistical analysis

All experiments were conducted with at least four biological replicates. The data are presented as the mean ± standard error. Statistical significance was determined using Student’s *t*-test (for two-group comparisons) or one-way ANOVA (or multi-group comparison) followed by Duncan’s multiple range test where appropriate. Statistical analyses were performed using R software (version 4.3.1), and significance levels were set at *p* < 0.05.

## Supplementary Information

Supplementary Material 1: Table S1 Formula of culture medium, Table S2 Primers used in this study.

## Data Availability

Data sharing is not applicable to this article, as no datasets were generated or analyzed during the current study.

## Abbreviations

TPS: Terpene synthases
FAC: Fatty acid‒amino acid conjugate
GPP: Geranyl diphosphate
FPP: Farnesyl diphosphate
GGPP: Geranylgeranyl diphosphate
IPP: Isopentenyl diphosphate
DMAPP: Dimethylallyl diphosphate
MVA: Mevalonate
MEP: 2-C-methyl-d-erythritol 4-phosphate
EBF: (*E*)-β-Farnesene
EAB: (*E*)-α-Bergamotene
DMNT: (3*E*)-4,8-Dimethyl-1,3,7-nonatriene
TMTT: (*E*, *E*)-4,8,12-Trimethyltrideca-1,3,7,11-tetraene
PTM: Post-translational modification
PEG: Polyethylene glycol
GLVs: Green leaf volatiles
CUT&Tag: Cleavage under targets and tagmentation
AmiRNAs: Artificial microRNAs
MeJA: Methyl jasmonate
eGFP: Enhanced green florescence protein
UBI: Ubiquitin
