# Supplementary material for: A maize protoplast transfection system for studying the biosynthesis of volatile terpenoids
Source: Crop Health. 2026 May 7;4(1):13. doi: 10.1007/s44297-026-00076-5 (PMC13153278; doi:10.1007/s44297-026-00076-5)
Supplement: Supplementary file 1 — Supplementary Material 1: Table S1 Formula of culture medium, Table S2 Primers used in this study. [file 44297_2026_76_MOESM1_ESM.docx]

**A maize protoplast transfection system for studying the biosynthesis of volatile terpenoids**

Jinfeng Qi^1,2,3^, Mengjing Li^1,2^, Zhonghua Hu^1,2^, Runsen Li^1,2^, Jing Li^1,3^, Mou Zhang^1,3^, Canrong Ma^1,3^, Jianqiang Wu^1,2,3^*

^1^Department of Economic Plants and Biotechnology, Yunnan Key Laboratory for Wild Plant Resources, Kunming Institute of Botany, Chinese Academy of Sciences, Kunming 650201, China

^2^CAS Center for Excellence in Biotic Interactions, University of Chinese Academy of Sciences, Beijing 100049, China

^3^State Key Laboratory of Plant Diversity and Prominent Crops, Beijing 100093, China

**Corresponding author**

Jianqiang Wu, email: [wujianqiang@mail.kib.ac.cn](mailto:wujianqiang@mail.kib.ac.cn)

ORCID: <https://orcid.org/0000-0002-7726-6216>

**Table S1 Formula of culture medium**

| **MMG buffer** | 4 mM MES (pH 5.7)  0.4 M mannitol  15 mM MgCl2 |
| --- | --- |
| **W5 solution** | 2 mM MES (pH 5.7)  5 mM KCl  125 mM CaCl_2_  154 mM NaCl |
| **N-Medium** | 2 mM MES (pH 5.7)  5 mM KCl  125 mM CaCl_2_  154 mM NaCl  10 mM MgCl  10 mM Sucrose  20 mM Glucose  4.43g/L MS |

**Table S2 Primers used in this study**

| ZmTPS10-F | ATGGATGCCACCGCCTTCCA |
| --- | --- |
| ZmTPS10-R | TTAGTAGAATAATGATATTG |
| ZmMYC2a-F | ATGAACCTGTGGACGGACGA |
| ZmMYC2a-R | TTACCTGCCCATGGCAGACC |
